# Impact of Arginine Nutrition and Metabolism during Pregnancy on Offspring Outcomes

**DOI:** 10.3390/nu11071452

**Published:** 2019-06-27

**Authors:** Chien-Ning Hsu, You-Lin Tain

**Affiliations:** 1 Department of Pharmacy, Kaohsiung Chang Gung Memorial Hospital, Kaohsiung 833, Taiwan; 2 School of Pharmacy, Kaohsiung Medical University, Kaohsiung 807, Taiwan; 3 Department of Pediatrics, Kaohsiung Chang Gung Memorial Hospital and Chang Gung University College of Medicine, Kaohsiung 833, Taiwan; 4 Institute for Translational Research in Biomedicine, Kaohsiung Chang Gung Memorial Hospital and Chang Gung University College of Medicine, Kaohsiung 833, Taiwan

**Keywords:** arginine, asymmetric dimethylarginine, citrulline, developmental origins of health and disease (DOHaD), glutamate, glutamine, nitric oxide, oxidative stress, pregnancy

## Abstract

By serving as a precursor for the synthesis of nitric oxide, polyamines, and other molecules with biological importance, arginine plays a key role in pregnancy and fetal development. Arginine supplementation is a potential therapy for treating many human diseases. An impaired arginine metabolic pathway during gestation might produce long-term morphological or functional changes in the offspring, namely, developmental programming to increase vulnerability to developing a variety of non-communicable diseases (NCDs) in later life. In contrast, reprogramming is a strategy that shifts therapeutic interventions from adulthood to early-life, in order to reverse the programming processes, which might counterbalance the rising epidemic of NCDs. This review presented the role of arginine synthesis and metabolism in pregnancy. We also provided evidence for the links between an impaired arginine metabolic pathway and the pathogenesis of compromised pregnancy and fetal programming. This was followed by reprogramming strategies targeting the arginine metabolic pathway, to prevent the developmental programming of NCDs. Despite emerging evidence from experimental studies showing that targeting the arginine metabolic pathway has promise as a reprogramming strategy in pregnancy to prevent NCDs in the offspring, these results need further clinical application.

## 1. Introduction

Arginine, a semi-essential amino acid, is a precursor in the synthesis of numerous molecules, such as nitric oxide (NO) and polyamines, which play decisive roles during pregnancy and fetal development [1]. Nutritional requirements for arginine can be met through dietary protein intake, de novo synthesis, and turnover of body proteins [2]. Markedly increased demands for arginine in pregnancy require greater dietary consumption to meet requirements [3]. Maternal malnutrition has significant consequences on offspring health. The developing fetus, if exposed to a suboptimal in utero environment, responds by developing adaptations that increase susceptibility to the development of a variety of adult-onset diseases, later in life. This concept has been consolidated as the Developmental Origins of Health and Disease (DOHaD) [4].

Non-communicable diseases (NCDs) are the number one cause of death worldwide [5]. NCDs are chronic diseases and nearly all of them can originate in early life [6]. As scientific knowledge emerges on the role of the DOHaD concept in the development of NCDs, evidence suggests that much more attention is needed on early-life interventions to curtail the increasing prevalence of NCDs [6]. Reprogramming is a strategy to reverse programming processes by changing the therapeutic interventions from the adult stage to the fetal/infantile stage, which precedes the onset of the clinical phenotype [7,8]. A growing body of evidence suggests the beneficial effects of supplemental arginine on human health [9,10]. Dietary arginine supplementation during pregnancy has started to gain importance as a reprogramming strategy to prevent adult-onset diseases [3,11,12].

Although there are many reviews discussing the biological roles of arginine, a literature focused on the impact of arginine on developmental programming of NCDs remains limited. For the purpose of this narrative review, electronic searches were performed in the database PubMed. The following keywords were searched—“arginine”, “nitric oxide”, “citrulline”, “glutamate”, “glutamine”, “developmental programming”, “pregnancy”, “mother”, “maternal”, “gestation”, “DOHaD”, “offspring”, “reprogramming”, and “dietary supplements.” Relevant free-access abstracts were identified and reviewed to determine appropriate studies. Suitable published articles in the English language were included, and no restrictions were applied to the dates of articles. Although there were a lot of papers relevant to arginine in pregnancy, only a few publications were focused on offspring outcomes.

This review highlights evidence for the metabolic fate of arginine, the role of arginine in normal and compromised pregnancy, the programming effects of arginine deficiency on offspring outcomes, and the targeting of the arginine pathway as a reprogramming strategy to prevent NCDs.

## 2. Biosynthesis and Metabolism of Arginine

### 2.1. De Novo Synthesis

**Figure 1:**
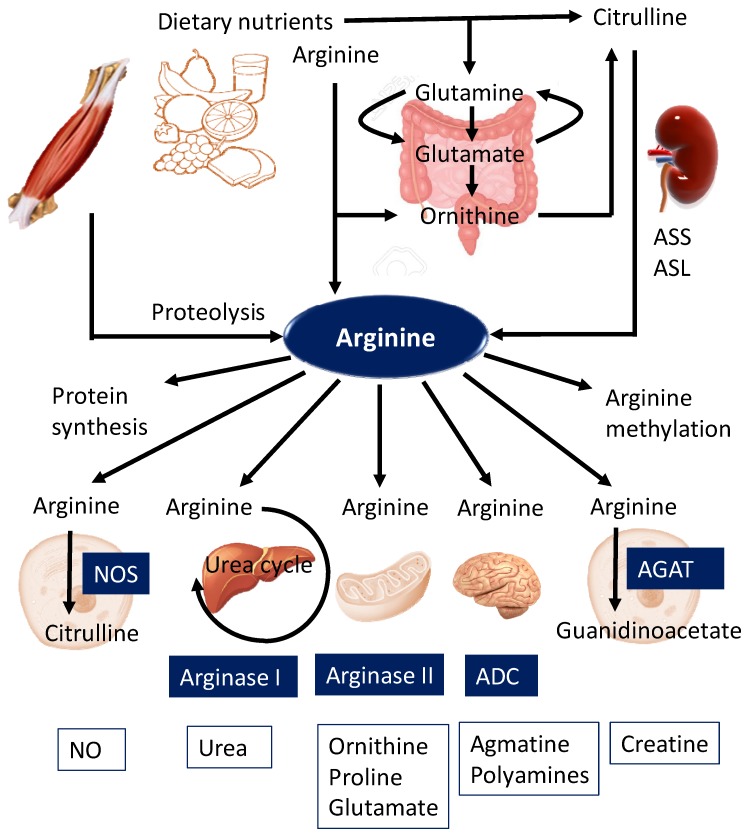
Biosynthesis and metabolism of arginine during physiological conditions. ASS—argininosuccinate synthetase; ASL—argininosuccinate lyase; NO—nitric oxide; NOS—nitric oxide synthase; ADC—arginine decarboxylase; AGAT—arginine:glycine amidinotransferase.

Sources of free arginine in humans are dietary intake (approximately 4–6 g of arginine per day), endogenous synthesis from citrulline (10–15% of the total arginine production), and protein turnover, which contribute to approximately 80% of the circulating arginine [1,2] (Figure 1). Dietary sources of arginine include meat, dairy products, eggs, nuts, and seeds. Endogenous de novo synthesis of arginine involves the conversion of citrulline to arginine via a two-step enzymatic process involving the enzymes argininosuccinate synthase (ASS) and argininosuccinate lyase (ASL) in the intestinal–renal axis [2,13]. As shown in Figure 1, citrulline is synthesized from glutamine, glutamate, and ornithine in the mitochondria of enterocytes, released into circulation, and is taken up primarily by kidneys for arginine synthesis. Additionally, citrulline can be converted into arginine in almost all cell types, including endothelial cells, enterocytes, macrophages, adipocytes, neurons, and myocytes [2].

### 2.2. Metabolism

Arginine is catabolized by five different groups of enzymes—(1) nitric oxide (NO) synthases (NOSs) for NO production; (2) arginase I as part of the urea cycle; (3) arginase II for the synthesis of ornithine, proline, and glutamate; (4) arginine decarboxylase (ADC) for agmatine generation in the brain and kidney; and (5) arginine:glycine amidinotransferase (AGAT) for the production of guanidinoacetate, the immediate precursor of creatine [2]. Through these catabolic pathways, arginine gives rise to NO and citrulline, urea, ornithine, proline, glutamate, agmatine, polyamines, guanidinoacetate, and creatine (Figure 1). Quantitatively, arginine is largely catabolized by the arginase pathway. Less than 2% of the metabolized arginine is utilized for NO production or polyamine synthesis [1]. Furthermore, arginine is involved in protein synthesis. Given that biochemical metabolic pathways of arginine are very complex, substrate competition between arginase and NOS is considered as a major factor contributing to intracellular arginine availability [14].

### 2.3. Transport

NO bioavailability depends on intracellular arginine concentrations [15]. Intracellular stores of arginine mainly come from de novo synthesis and turnover of intracellular proteins. On the other hand, arginine can be used as the substrate for several metabolic pathways. A previous report demonstrated that only 1.5% of arginine enters the NOS pathway [16]. Despite the substantial intracellular stores of arginine, extracellular arginine is rate limiting for NO production, a phenomenon called the “arginine paradox” [15].

So far, arginine transport into or out of the cell can be mediated by at least 4 different cationic amino acid transporter (CAT) proteins—CAT-1, -2A, -2B, and -3. CAT-1 is the predominant arginine transporter ubiquitously expressed in almost all types of cells [17]. Co-localization of CAT-1 and endothelial NOS (eNOS) in caveolae, in part, explains the “arginine paradox”, which is related to the phenomenon that in certain disease states, eNOS requires an extracellular supply of arginine, regardless of whether there is adequate intracellular arginine concentrations [15]. CAT2A, a low-affinity splice variant, exhibits a distinct expression localized mainly in the liver. CAT-2B expression can be induced in many cell types by treatment with inflammatory cytokines, often together with inducible NOS (iNOS) [17]. CAT-3 is widely expressed during embryonic development, while it appears to be confined to the central neurons in adult rodents [17]. As arginine shares the same transport system with other cationic amino acids, such as lysine and ornithine, arginine uptake via CATs can be competitively inhibited by these amino acids.

**Figure 2:**
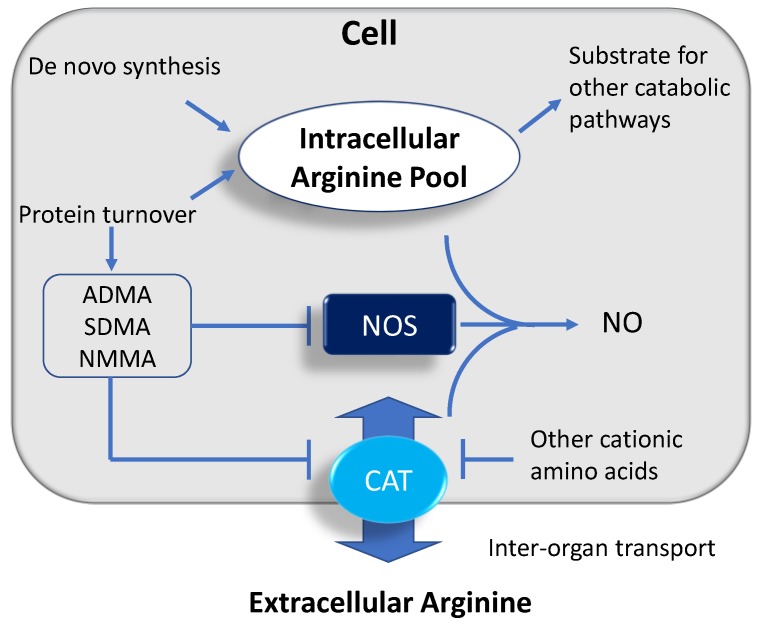
Schema outlining the synthesis, metabolism, and transfer of arginine to maintain intracellular arginine concentrations for NOS to produce NO. NO—nitric oxide; NOS—nitric oxide synthase; NMMA—N^G^ monomethyl-L-arginine; ADMA—asymmetric dimethylarginine; SDMA—symmetric dimethylarginine; CAT—cationic amino acid transporter.

In addition to a connection between arginine transport and intracellular arginine availability, the competition between arginine and its isomer asymmetric dimethylarginine (ADMA) for NOS, also plays a key role on NO production [18]. The biochemical pathways related to the synthesis, metabolism, and transport of intracellular arginine and their relationships to NO production are illustrated in Figure 2.

### 2.4. Arginine Methylation

The nitrogen atom of arginine within proteins can be post-translationally modified to contain methyl groups, a process termed arginine methylation [19]. Protein-incorporated ADMA is formed by post-translational methylation—two methyl groups are positioned on one of the terminal nitrogen atoms of the guanidino group of arginine in proteins, by a family of protein arginine methyltransferases (PRMTs) [19]. Symmetric dimethylarginine (SDMA), with one methyl group placed on each of the terminal guanidine nitrogens, is a structural isomer of ADMA [20]. Free ADMA and SDMA are released following proteolysis. Free ADMA and SDMA share common CATs with arginine to move into or out of cells [21]. Both ADMA and SDMA are well-known for their inhibition of NO production [21,22]. ADMA, in pathological concentrations, competes with arginine to inhibit NOS activity, leading to a reduction of NO. On the other hand, SDMA does not directly inhibit NOS but is a competitive inhibitor of arginine transport [23]. Dimethylarginine dimethylaminohydrolase-1 (DDAH-1) and -2 (DDAH-2) have been reported to metabolize ADMA to citrulline and dimethylamine. Alanine-glyoxylate aminotransferase 2 (AGXT2), a mitochondrial aminotransferase expressed primarily in the kidney, can metabolize ADMA as well as SDMA. In addition to dimethylarginines, another arginine analog is N^G^ monomethyl-l-arginine (NMMA). In our body, plasma levels of NMMA are much lower than those of ADMA and SDMA. Like ADMA, NMMA is a competitive NOS inhibitor [22]. However, there is very little information available on the pathophysiological role of NMMA in human health.

## 3. Arginine Nutrition in Pregnancy and Fetal Development

Maternal nutrition is vital to placental and fetal development during gestation. By serving as a precursor for the synthesis of biologically important substances, arginine plays a decisive role in nutrition and metabolism [2,3]. A corpus of evidence is emerging showing that arginine plays a crucial role in reproduction, fetal development, wound healing, maintenance of tissue integrity, and immune function, as well as treatment of diseases in pregnancy [1,2,3,9,10,11]. Maternal plasma arginine concentrations were found to be lower in pregnancies complicated by intrauterine growth retardation (IUGR) [24]. Arginine can directly activate p70 S6 kinase and phosphorylation of 4E-BP1 through the mechanistic target of the rapamycin (mTOR) signaling pathway, to stimulate protein synthesis [25]. Arginine is also required for the urea cycle to remove ammonia from the liver and blood. High levels of ammonia are detrimental to the developing fetus through the induction of oxidative stress, increase in intracellular pH, reduction of ATP production, and decrease in utero–placental blood flow and nutrient transport [26].

Arginine is a common substrate for NO and polyamines (putrescine, spermine, and spermidine). It is well-known that NO and polyamines are both crucial for fertilization, implantation, embryonic development, and placental angiogenesis [27]. Additionally, NO is an endothelium-derived relaxing factor. NO is essential to the regulation of placental–fetal blood flow. Thus, NO might play a crucial role in maintaining adequate transfer of nutrients from mother to fetus. Likewise, polyamines regulate numerous cellular functions from gene expression to protein synthesis, which performs embryo/fetus proliferation, growth, and differentiation [28]. Furthermore, NO and polyamines are key regulators of angiogenesis. Impaired angiogenesis was reported in eNOS knockout mice [29]. In vivo knockdown of eNOS in ovine conceptuses stunted morphological development and subsequently reduced levels of arginine and polyamines in conceptus tissues [30].

On the other hand, maternal plasma ADMA levels are reduced in the early stage of gestation but increase as the gestational age increases [31]. In early pregnancy, the reduction in ADMA and the concomitant increase in NO can aid in hemodynamic adaptation and uterine relaxation, to avoid disturbed intrauterine growth of the fetus. On the contrary, NO-induced uterine relaxation in late pregnancy can be antagonized by physiologically increased ADMA levels, to help prepare the uterine muscle fibers for the higher contractile activity that is necessary for successful delivery.

To sum up, these observations indicate that complex regulation of different elements in the arginine metabolic pathway during pregnancy is required to ensure successful pregnancy and offspring outcomes.

## 4. Impaired Arginine Metabolic Pathway in Compromised Pregnancy and Fetal Programming

Protein restriction during pregnancy is linked to IUGR, reduced postnatal growth, and a variety of adult-onset diseases [32,33,34]. Maternal high protein intake is also related to IUGR and can cause fetal or neonatal death due to ammonia toxicity [26]. High protein intake in gestation is harmful due to excesses and imbalances of amino acids. With high maternal concentrations of amino acids, competition for their transporters leads to reduced placental transport and umbilical uptake of amino acids. There is growing interest in the impact of certain amino acids on pregnancy and offspring outcomes. One of these amino acids is arginine, as its derived metabolites play a key role in fetal growth and development [3].

Arginine metabolism is altered in compromised pregnancy, with regard to both its synthesis and its catabolism. This can result in a disruption of the fetal development, resulting in fetal programming and an increased risk for developing adult-onset diseases. Each adverse condition have been discussed here.

### 4.1. Preeclampsia

Adverse pregnancy outcomes such as preeclampsia, preterm labor, stillbirth, and fetal growth restriction continue to be major causes of morbidity and mortality for mothers and infants [35]. Preeclampsia is a major cause of IUGR, premature labor, and maternal death all over the world. Preeclampsia is a syndrome of the second half of pregnancy, characterized by hypertension, proteinuria, and edema. In humans, plasma levels of arginine and placental eNOS abundance are reduced in preeclamptic compared to healthy pregnant women [36]. Evidence from animal models demonstrated that arginine deficiency in the preeclampsia placenta decreased NO and increased superoxide formation, resulting in NO deficiency and excessive formation of peroxynitrite [37]. Additionally, ADMA levels were reported to increase in the preeclamptic compared to healthy pregnant women [38], even before the development of preeclampsia [39]. These observations suggest that elevated ADMA concentrations are associated with an increased risk of developing preeclampsia in pregnant women.

Conversely, arginine supplementation has shown benefits with respect to preeclampsia. A meta-analysis including seven studies with 916 patients reported that arginine supplementation could reduce preeclampsia incidence and lower diastolic blood pressure (BP) by 4.86 mmHg [40]. In a rat model of preeclampsia induced by N^G^-nitro-L-arginine methyl ester (L-NAME), intravenous arginine treatment (21 mg/kg per day from gestational day 16 through delivery) reversed hypertension, proteinuria, renal glomerulus injury, and IUGR [41]. Additionally, preeclamptic women receiving oral arginine supplementation at a dose of 3 g per day from admission through delivery were reported to improve maternal and offspring outcomes. These benefits included increased NO synthesis, reduced BP, prolonged pregnancy, improved fetal wellbeing, and enhanced fetal growth [42].

### 4.2. Gestational Diabetes Mellitus

Gestational diabetes mellitus (GDM) is the most common metabolic perinatal complication and it is a common maternal condition causing developmental programming of various adult-onset diseases [43]. GDM is a syndrome characterized by hyperglycemia, glucose intolerance, abnormal regulation of vascular tone, and endothelial dysfunction [43]. GDM is associated with endothelial dysfunction attributed to a dysregulated endothelial arginine/NO signaling pathway [44]. An increase in the level of arginine in the umbilical artery and veins of women with GDM as compared to normal pregnant women was reported [45]. Additionally, human umbilical vein endothelial cells from gestational diabetic pregnancies showed increased arginine transport via CATs and increased NO synthesis by eNOS [46]. These results suggest that GDM is associated with an upregulation in the endothelial arginine/NO pathway. On the other hand, previous studies demonstrated that the ADMA level was elevated in women with GDM and was positively correlated with glucose levels [47,48]. Even when the arginine/NO pathway in the fetoplacental vasculature was upregulated, NO bioavailability might have been reduced due to the increased ADMA level seen in GDM [49]. Furthermore, arginase activity was positively correlated with glucose intolerance and was higher in cord blood of GDM mothers, compared to the control group [50]. As the arginase pathway limits the arginine availability for NO synthesis, this finding suggests a dysregulated arginine metabolic pathway is also involved in the pathogenesis of GDM.

### 4.3. Intrauterine Growth Retardation

IUGR is defined as a significant reduction in the fetal growth rate resulting in a birth weight below the tenth percentile for gestational age [51]. It is well-known that IUGR is associated with an increased risk of cardiovascular, metabolic, and neurological diseases later in life [52].

NO is a vasodilator and angiogenic factor. NO deficiency impairs placenta angiogenesis, protein synthesis in the placenta, and uteroplacental circulation in pregnancies complicated by IUGR [53]. Additionally, reduced placental and fetal growth are associated with reductions in placental polyamine transport, as well as concentrations of polyamines in gestating dams [54]. Conversely, by regulating syntheses of NO and polyamines, arginine supplementation was reported to stimulate placental growth and the transfer of nutrients from mother to fetus, to promote fetal growth and development [3]. During the intrauterine period, most disorders do not directly program the fetus but instead first affect placental development and function. Thus, the placenta acts as a nutrient sensor, modifying nutrient and hormone availability to fetoplacental tissues, in response to environmental challenges [55].

In a protein restriction-induced IUGR rat model, reduced expression of CAT-1 was related to decreases in fetal and placental weight [56]. In pigs, IUGR had permanent negative impacts on organ structure, preweaning survival, postnatal growth, lifetime health, and the onset of adult diseases [57]. However, supplementation of drinking water with 0.2% or 2% arginine prevented hypoxia-induced IUGR in rats [58]. Likewise, arginine supplementation was reported to improve uteroplacental circulation and infant weight at birth in women presenting IUGR [59].

### 4.4. Prematurity and Low Birth Weight

Preterm birth is the leading cause of neonatal and childhood morbidity [60]. A low birth weight might be the result of a premature birth. It can also be caused by an IUGR. Epidemiological studies show a link between low birth weight and chronic diseases in adults [61]. Reduction in the umbilical vein plasma levels of arginine and its derived amino acids were reported in low birth weight newborns in humans [62], pigs [63], and rats [64].

In a mouse model, inhibition of NO by L-NAME caused preterm delivery, which was reversed by infusion of sodium nitroprusside (a NO donor) [65]. In humans, women with the preterm onset of uterine contractions received intravenous infusion of arginine, resulting in a reduction of spontaneous uterine contractility [66]. Similarly, another report showed that oral arginine supplementation improved fetoplacental blood flow distribution in pregnant women with threatened preterm labor [67]. These observations suggest that arginine supplementation might have a beneficial effect on preterm birth.

### 4.5. Developmental Programming of Adult-Onset Diseases

So far, an arginine-deficient diet in pregnancy has been understudied with regards to adult offspring health. However, a low protein diet in pregnancy has been extensively used to explore the pathogenesis of developmental programming of various adult-onset diseases [33]. In rodents, a maternal low protein diet induced hyperglycemia, glucose intolerance, insulin resistance, obesity, and hypertension in adult offspring [33,68,69]. Similarly, a maternal low protein diet was reported to program metabolic syndrome-related phenotypes in adult offspring in other species, like pigs, sheep, and cows [26,69].

NO is an important product of arginine. Adult rat offspring born of dams exposed to the NOS inhibitor L-NAME in pregnancy developed hypertension, proteinuria, and kidney disease [41,70]. As we reviewed elsewhere, another NOS inhibitor ADMA is also involved in the developmental programming of hypertension [71]. Conversely, early reprogramming interventions targeting the ADMA-related NO deficiency was reported to prevent the development of programmed hypertension in both genetic and developmentally programmed hypertension models [71]. These findings suggest an impaired arginine metabolic pathway during pregnancy increases the risk of developing many chronic diseases in later life.

## 5. Targeting the Arginine Metabolic Pathway to Prevent Adult-Onset Diseases

Reprogramming strategies include nutritional intervention, pharmacological therapy, and lifestyle modification. These approaches aim to reverse the early-life disorders that induced programmed development and consequent adverse outcomes. Thus, adverse programmed processes during a compromised pregnancy can be prevented or at least reduced by appropriate reprogramming interventions. Certain nutritional interventions in gestation and lactation were reported to be beneficial against some adult-onset diseases [12,34].

We restricted this review to only nutritional interventions during pregnancy or lactation periods, as there are reprogramming strategies to prevent diseases of developmental origins in all sorts of animal models, which are listed in Table 1 [41,70,72,73,74,75,76,77,78,79,80,81,82,83,84]. The most commonly used animal species include rats, pigs, and sheep. Both male and female animals were examined. The sample size in these reported studies varied from 5 to 18. Since arginine precursors, such as citrulline, glutamine, and glutamate, could be an alternative to obtaining the benefits provided by arginine, such nutritional interventions with regard to the arginine metabolic pathway were recruited.

### 5.1. Arginine

Arginine supplementation has been tested in many human diseases and experimental animals as a way to improve NO bioavailability [2,85]. Nevertheless, the benefits of arginine from human trials remain inconclusive [10]. Arginine supplementation varying between 3 and 100 g/day with the duration ranging from 3 days to 18 months have been used in clinical studies. Single doses exceeding 9 g and a dosing regimen of over 30 g/day have been associated with gastrointestinal upset [86].

Despite the studies described above, little is known about the reprogramming effects of arginine supplementation during pregnancy on the offspring outcome. In rats, maternal arginine supplementation improved IUGR born of dams exposed to hypoxia [58] and L-NAME [41]. In an ovine caloric restriction model, arginine supplementation starting from early to middle gestation appeared beneficial by improving IUGR [33,68,69]. In swine, arginine supplementation starting from early gestation (day 30) to delivery, resulted in increased litter birth weight [75]. Arginine supplementation during late gestation (days 90 to 114) also increased the birth weights of offspring [76]. Despite post-weaning, arginine supplementation have been reported to improve hypertension, insulin sensitivity, and beta cell function in offspring rats [87,88], currently the reprogramming effects of arginine supplementation in pregnancy other than IUGR have not been fully examined.

### 5.2. Citrulline

Oral citrulline supplementation is a potential way to raise plasma arginine concentration as it can bypass hepatic metabolism and can allow renal conversion to arginine [89]. Of note, citrulline has a limited degradation in the placenta, being efficiently transferred from the mother to the fetus in favor of fetal development [90]. In humans, circulating arginine concentrations peak after approximately 1–2 h, following oral citrulline supplementation [90]. Citrulline supplementation as a single oral dose ranging between 2 and 15 g is safe and well-tolerated in healthy adults [91]. Although recent evidence suggests beneficial effects of citrulline supplementation on cardiometabolic health [92], the long-term effects of citrulline supplementation in gestation on offspring outcomes remain unclear.

Citrulline supplementation was also used as a reprogramming intervention in a rat model of maternal low protein diet to prevent IUGR [77,78]. Maternal citrulline supplementation protected adult rat offspring against hypertension and kidney disease, in several models of developmental programming, including maternal caloric restriction [79], streptozotocin-induced diabetes [80], prenatal dexamethasone exposure [81], and maternal L-NAME exposure [70,82]. Furthermore, perinatal citrulline supplementation can restore NO bioavailability to prevent the transition of prehypertension to hypertension in spontaneously hypertensive rats [83]. In a maternal NO depletion induced by the L-NAME model, programmed hypertension prevented by maternal citrulline supplementation was relevant to an alteration of renal transcriptome, with more than 300 genes [82]. As nutrients do not drive their programming effect independently from each other, a key question for future research is—what are the nutrient–nutrient interactions and nutrient–gene interactions that can affect the programming power on offspring outcomes?

### 5.3. Glutamate and Glutamine

Glutamate and glutamine are highly abundant amino acids. Both amino acids are precursors for arginine synthesis. Since there is an increased need for them during fetal growth, glutamate and glutamine are considered as conditionally essential during pregnancy [93]. The N-carbamoylglutamate (NCG) molecule can activate carbamoylphosphate synthetase, a key enzyme in the process of arginine synthesis in enterocytes from carbamoyl phosphate and ornithine [94]. In ruminants, dietary NCG supplementation was reported to increase the endogenous synthesis of arginine, as NCG was not affected by ruminal metabolic degradation [95]. NCG supplementation during pregnancy was shown to improve IUGR in sheep [72] and pigs [76]. In an ovine model, glutamine supplementation during late gestation (days 109 to 132) also improved IUGR induced by maternal alcohol exposure [79]. Still, long-term reprogramming effects of glutamate and glutamine on offspring health in later life remain largely unknown.

### 5.4. Others

There are other potential interventions related to the arginine metabolic pathway by which arginine bioavailability could be increased, such as arginase inhibition and ADMA-lowering agents. First, active arginase can reduce the supply of arginine needed for the production of NO by NOS [96]. Thus, arginase inhibitor has shown beneficial effects via restoration of NO in several human diseases. A previous study showed that arginase activity was increased and eNOS activity was decreased in IUGR umbilical and placental vessels [97]. In a rat model of IUGR, arginase upregulation and eNOS uncoupling were related to hypertension in adult offspring [98]. These findings suggest that arginase inhibitor might serve as a potential reprogramming strategy to restore the arginine-NO pathway in the prevention of adult-onset diseases later in life. Next, as intracellular arginine and ADMA competes for NOS binding, ADMA-lowering interventions could be another approach to restore the impaired arginine-NO pathway. So far, a specific ADMA-lowering agent has not yet been discovered [21]. As we reviewed elsewhere, a number of studies have indicated that pravastatin, resveratrol, aminoguanidine, farnesoid X receptor agonists, salvianolic acid A, vitamin E, melatonin, metformin, pioglitazone, probucol, N-acetylcysteine, and aliskiren can increase the activity or expression of DDAH, and thereby reduce the ADMA levels [71,99]. Additionally, telmisartan, glucagon-like peptide-receptor agonist, and rosuvastatin can decrease PRMT-1 expression and reduce ADMA. As PRMTs regulate ADMA production and as DDAHs regulate its catabolism, the discovery of specific PRMT inhibitors or DDAH activators might represent potential reprogramming strategies to improve arginine bioavailability. Additional studies exploring the reprogramming effects of these ADMA-lowering agents in the prevention and treatment of adult-onset diseases are enormously warranted.

## 6. Conclusions

Although advances in research indicate that arginine plays a key role in pregnancy and offspring outcomes, there is much more to be learned. Given the complexity of arginine metabolic pathways, elucidation of the reprogramming effects of different arginine-related elements in various animal models is required to ensure their successful clinical translation. Targeting the arginine metabolic pathways as a reprogramming strategy against the developmental programming of various NCDs remains a promising challenge in the DOHaD field and will become even more important in light of the rising epidemic of NCDs. This review has provided an overview on reprogramming strategies excepting arginine, which are related to the arginine metabolic pathway, including citrulline, glutamine, and glutamate. It is clear that a better understanding of the type of arginine-derived molecules, dose of supplement, and therapeutic duration in pregnancy are needed before the mother and child can benefit from reprogramming strategies targeting the arginine metabolic pathway.

## Figures and Tables

**Table 1:** Reprogramming strategies targeting the arginine metabolic pathway to prevent developmental programming of adult-onset diseases in animal models.

Interventions	Animal Models	Species/ Gender	Age at Measure	Reprogramming Effects	Ref.
Arginine					
0.2 or 2% in water from gestational day 1 to 21	Hypoxia exposure	Wistar rat/M and F	Gestational day 21	Improved IUGR	[58]
21 mg/kg daily from gestational day 16 to delivery	Maternal L-NAME exposure	SD rat/M and F	Birth	Improved IUGR	[41]
10 g/day in diet from gestational day 35 to 110	Maternal 50% caloric restriction	Ovine/M and F	Gestational day 110	Improved IUGR	[72]
155 μmol/kg i.v. 3 times daily from gestational day 60 to delivery	50% caloric restriction	Ovine/M and F	Birth	Improved IUGR	[73]
180 mg/kg once daily from gestational day 54 to delivery	40% caloric restriction	Ovine/M and F	Birth	Improved IUGR	[74]
1% in diet from gestational day 30 to 114	Spontaneous IUGR	Swine/M and F	Birth	Improved IUGR	[75]
1% in diet from gestational day 90 to delivery	Spontaneous IUGR	Swine/M and F	Birth	Improved IUGR	[76]
Citrulline					
2 g/kg/day in water in pregnancy	Low protein diet	SD rat/M and F	Gestational day 21	Prevented IUGR	[77]
2 g/kg/day in water in pregnancy	Low protein diet	SD rat/M and F	Birth	Prevented IUGR	[78]
2.5 g/L of water in pregnancy and lactation	Maternal 50% caloric restriction	SD rat/M	12 weeks	Prevented low nephron number and renal dysfunction	[79]
2.5 g/L of water in pregnancy and lactation	Streptozotocin-induced diabetes	SD rat/M	12 weeks	Prevented hypertension and kidney injury	[80]
2.5 g/L of water in pregnancy and lactation	Prenatal dexamethasone exposure	SD rat/M	12 weeks	Prevented hypertension	[81]
2.5 g/L of water in pregnancy and lactation	Maternal L-NAME exposure	SD rat/M	12 weeks	Prevented hypertension	[70,82]
2.5 g/L of water from gestational day 7 to postnatal week 6	Genetic hypertension model	SHR/M and F	50 weeks	Prevented hypertension	[83]
Glutamate (N-carbamoylglutamate)					
2.5 g/day in diet from gestational day 35 to 110	Maternal 50% caloric restriction	Ovine/M and F	Gestational day 110	Improved IUGR	[72]
0.1% in diet from gestational day 90 to delivery	Spontaneous IUGR	Swine/M and F	Birth	Improved IUGR	[76]
Glutamine					
100 mg/kg i.v. 3 times daily from gestational day 109 to 132	Maternal alcohol exposure	Ovine/M and F	Gestational day 132	Improved IUGR	[84]

Studies tabulated according to animal models, species, and age at measurement. SD—Sprague–Dawley rat; M—male; F—female; L-NAME—N^G^-nitro-L-arginine-methyl ester.
